# Validation of the humour styles questionnaire in healthcare professionals

**DOI:** 10.1002/nop2.1528

**Published:** 2022-12-20

**Authors:** Miriam Leñero‐Cirujano, Juan Ignacio Torres‐González, Héctor González‐Ordi, Jacinto Gómez‐Higuera, Mª Nieves Moro‐Tejedor

**Affiliations:** ^1^ Department of Nursing Universidad Alfonso X El Sabio Madrid Spain; ^2^ Department of Nursing Research Nursing Group of Instituto de Investigación Sanitaria Gregorio Marañón (IiSGM) Madrid Spain; ^3^ Department of Nursing Hospital Universitario Clínico San Carlos Madrid Spain; ^4^ Department of Psychology Universidad Complutense University Madrid Spain; ^5^ Department of Nursing Universidad Complutense University Madrid Spain

**Keywords:** health professionals, humour, nursing, questionnaire, validation

## Abstract

**Aim:**

The aim of this study is to determine the reliability and validity of the Humour Styles Questionnaire (HSQ) in a sample of Spanish healthcare professionals.

**Design:**

A cross‐sectional study.

**Methods:**

The version of HSQ translated into Spanish by Cayssials and Pérez was used to validate on a sample of healthcare professionals (N = 250). The reliability analysed the Crombach's *α* coefficient and Pearson's correlation coefficient between the factors and the total scale score. The Exploratory Factor Analysis was carried out with Kaiser's criteria for the extraction of factors with Varimax rotation.

**Results:**

HSQ in this study sample reproduced the similar structure of the original version with four factors (affiliative, self‐enhancing, aggressive and self‐defeating humour). These factors explained 44.46% of the total variance and Cronbach's ranged from 0.64–0.79. Global HSQ scale reliability was 0.82.

**Conclusion:**

The HSQ is a valid and reliable instrument for assessing humour in healthcare professionals.

## 1 INTRODUCTION

The conceptualisation of humour is still a challenge for theorists today, due to its multidimensional character (Martin, 2001). One of the first and most precise contemporary definitions of this construct was that of Ruch (Ruch, 1998) distinguishing humour from sense of humour. The former would refer to a concrete situation that is qualified as funny or to specific material that is appreciated as humorous, while the latter would refer to a stable individual quality in the person. Martin (2001) posited a sense of humour as everything a person does and says that is perceived as humorous and tends to make people laugh, involving those mental processes that are aimed at creating and perceiving funny stimuli and their respective affective response. The author further differentiated four styles of humour, affiliative humour, self‐enhancing humour, aggressive humour and self‐defeating humour. He considered the first two as types of humour that are potentially beneficial for well‐being, while the latter are potentially harmful. Affiliative humour would relate to telling jokes or funny stories to make others laugh, joke or be amused without malicious intent in order to entertain and facilitate social relationships. Self‐enhancing humour would relate to coping with difficult circumstances through a humorous view of the world. Aggressive humour, with sarcasm, ridicule, irony or mockery as a means of criticizing or manipulating others. Self‐defeating humour, with denigration, belittling or ridicule of oneself in order to attract the attention of others (Martin et al., 2003; Mendiburo & Páez, 2011).

According to literature, affiliative and self‐enhancing humour act as health‐promoting humour styles and they were overall positively correlated with mental health. Self‐defeating humour was overall negatively correlated with mental health. On the contrary, aggressive humour was overall unrelated with mental health. Fostering specific humour styles may be beneficial for mental health (Schneider et al., 2018). These humour styles are assessed in the Humour Style Questionnaire (HSQ) designed by Martin et al. (2003). It is one of the most recommended questionnaires because of its multidimensional nature and its predictive value in measuring aspects of psychosocial well‐being. This instrument has been translated into more than 30 languages (Martin & Kuiper, 2016).

Healthcare professionals have a high workload and a lot of stress, which often lead to states that are difficult to manage, especially in the current pandemic situation (Danet, 2021). In this context, humour could act as a strategy for psychic and emotional control by allowing the professional to view daily and work‐related events from a more optimistic perspective (Vaz de Almeida & Nunes Health, 2020). The growing importance of humour lies in the potential benefits attributed to it in the scientific literature, both mental, emotional, physical and social. In the therapeutic relationship, humour has been shown to improve communication with the patient and the coping with difficult situations, helping to create a climate of trust and confidence (Birkelund & Larsen, 2013; Haydon & van der Riet, 2014; Leñero, 2015; Sousa et al., 2019). In the work environment, it improves the working atmosphere, favours relationships between colleagues, promotes teamwork, increases motivation and job satisfaction and reduces job burnout and stress, resulting in a higher quality of care (Bartzik et al., 2021; Brender‐Ilan & Reizer, 2021; Chen & Ayoun, 2019; Cheng et al., 2021; Mesmer‐Magnus et al., 2018; Vaz de Almeida & Nunes Health, 2020).

## 2 BACKGROUND

The English version of the HSQ has shown adequate reliability and validity results (Martin et al., 2003). It has been translated into other language versions such as the Spanish version (Cayssials & Pérez, 2005). In general Spanish population, the psychometric behaviour of this measure has been previously studied (Torres‐Marín et al., 2018) and successfully administered in Spanish health professionals (Navarro‐Carrillo et al., 2020). However, the reliability and validity of the instrument has not been tested in this population. It's important to remember that this population, due to the type of work they do, the workload and the pressure to which they are subjected, is more exposed to psychological problems such as stress, anxiety, depression and others (Arrogante & Aparicio‐Zaldivar, 2020; Dutheil et al., 2019), that influence the type of humour they use. In line with this, research suggests that the humour is influenced by social and cultural factors (Jiang et al., 2019; Jiang et al., 2020; Kuiper et al., 2010; Mendiburo & Páez, 2011; Yue et al., 2016), so the reliability and validity of the scale could vary according to the population. Considering the available scientific evidence on humour shows important benefits in healthcare professionals, this study aimed to determine the reliability and validity of the HSQ scale in a sample of Spanish healthcare professionals.

## 3 METHOD

### 3.1 Study design

This study employed a cross‐sectional design which a psychometric evaluation.

### 3.2 Participants

Data were collected from healthcare professionals of the “REDACTED” between June 2019 and December 2019. The inclusion criteria were to be healthcare professionals and to work in the hospital at the moment to complete the scale. The questionnaires less than 80% completed were excluded from this study.

Based on the previous works, it was determined that a minimum sample size would include 5–10 times the number of items was appropriate for a stable test of the reliability and validity of the measurement tool including factor analysis (Kline, 1994; Nunnally & Bernstein, 1994). At least 160 participants were needed to evaluate the psychometric properties of HSQ 32‐item. 800 questionnaires were delivered. The participants completed the demographic information questionnaire and the HSQ scale. People who agreed to participate in the study voluntarily after informing them of the purpose and basic method of participation in the study were included. Recruitment was carried out in the different randomized functional units by nursing supervisors. Before data collection, we carried out several informative sessions.

### 3.3 Measures

The original English version of the HSQ by Martin is made up of a 32‐item questionnaire grouped in four eight‐item correlated dimensions related to different humour styles: adaptive‐positive and beneficial humour (affiliative and self‐enhancing humour) and maladaptive‐negative and detrimental humour (aggressive and self‐defeating humour) (Martin et al., 2003). All items were answered using a 7‐point Likert type scale (from 1 = strongly disagree to 7 = strongly agree). High scores for each dimension indicate high levels for that style of humour.

Before the study, we contacted via e‐mail with the author to obtain his consent to use his scale and to carry out the validation process in health professionals. We used the Spanish version adapted in Argentine population by Cayssials and Pérez (2005). In this validation study, the HSQ structure explained 37.00% of the variance and a reliability of 0.75.

We included socio‐demographic information in the administered questionnaire: gender, age and professional category.

### 3.4 Statistical analysis

Demographic characteristics and scale scores were presented using descriptive statistics. Qualitative variables were described by frequencies and percentages. Quantitative variables were described with mean values and standard deviations.

Structural validity was measured through exploratory factor analysis (EFA). Principal components analysis was used to determine the construct validity of the scale. To determine suitability for EFA, the Kaiser‐Meyer‐Olkin (KMO) value was used to measure sampling adequacy. Bartlett's test for sphericity, factor loading and sampling adequacy measure were used to determine item adequacy. Standards for loading values vary between 0.30–0.50, but in this study, items with communality and loading value size less of 0.30 were removed (Lloret‐Segura et al., 2014). Factors with an eigenvalue greater than 1.0 were kept as common factors. The factorization of the correlation matrix was determined by the KMO test >0.70 was acceptable and statistically significant in the Bartlett sphericity test (Fabrigar et al., 1999).

Reliability was measured with the Cronbach's alpha coefficient of the total simple. A Cronbach's alpha coefficient higher than 0.7 was considered acceptable. The Pearson's correlation coefficient is used to measure the association between factors and total scale score. A Pearson correlation coefficient of 0.7 or high was considered acceptable (Carvajal et al., 2011).

The SPSS Statistics 24 for Windows were used to analyse the obtained data (IBM Corp., 2016). Statistical significance was set at *p* < 0.05.

## 4 RESULTS

### 4.1 Participants' characteristics

Table 1 presents the descriptive statistics for the demographic characteristics of the participants. Of the 800 questionnaires delivered, 250 questionnaires were filed, for a response rate of 31.25%. All respondents were aged between 19 and 61 years, with a mean age of 40.61 years (11.40). The data showed that 87.60% were female and 56.40% nurses.

**TABLE 1:** Socio‐demographic information

Variables	
Age (Mean, SD)	40.61 (11.40)
Gender, *N* (%)	
Female	219 (87.60)
Male	31 (12.40)
Professional category, *N* (%)	
Doctor	10 (4.00)
Nurse	141 (56.40)
Auxiliary Nursing	95 (38.00)
Others (Central's services)	4 (1.60)

Abbreviations: N, sample; SD, standard deviation.

### 4.2 Item analysis

Based on the result of the items' response rate, no items presented a percentage greater than 90% in a particular value. Only two items (item 23 and item 27) showed a percentage more than 80% but less than 90% in the first option (Table 2).

**TABLE 2:** Distribution of the HSQ responses

	Likert scale						
Items	1	2	3	4	5	6	7
1	1 (0.4%)	20 (8.0%)	15 (6.0%)	14 (5.6%)	18 (7.2%)	82 (32.8%)	100 (40.0%)
2	6 (2.4%)	13 (5.2%)	14 (5.6%)	29 (11.6%)	54 (21.6%)	60 (24.0%)	74 (29.6%)
3	187 (74.8%)	38 (15.2%)	8 (3.2%)	8 (3.2%)	5 (2.0%)	2 (0.8%)	2 (0.8%)
4	160 (64.0%)	48 (19.2%)	13 (5.2%)	9 (3.6%)	7 (2.8%)	7 (2.8%)	6 (2.4%)
5	13 (5.2%)	27 (10.8%)	17 (6.8%)	74 (29.6%)	47 (18.8%)	53 (21.2%)	19 (7.6%)
6	32 (12.8%)	25 (10.0%)	20 (8.0%)	41 (16.4%)	53 (21.2%)	48 (19.2%)	31 (12.4%)
7	127 (50.8%)	82 (32.8%)	17 (6.8%)	18 (7.2%)	4 (1.6%)	2 (0.8%)	‐
8	94 (37.6%)	31 (12.4%)	30 (12.0%)	32 (12.8%)	29 (11.6%)	16 (6.4%)	18 (7.2%)
9	2 (0.8%)	15 (6.0%)	16 (6.4%)	52 (20.8%)	31 (12.4%)	80 (32.0%)	54 (21.6%)
10	12 (4.8%)	19 (7.6%)	9 (3.6%)	56 (22.0%)	60 (24.0%)	53 (21.2%)	42 (16.8%)
11	159 (63.6%)	49 (19.6%)	13 (5.2%)	19 (7.6%)	5 (2.0%)	4 (1.6%)	1 (0.4%)
12	58 (23.2%)	38 (15.2%)	18 (7.2%)	43 (17.2%)	35 (14.0%)	43 (17.2%)	15 (6.0%)
13	‐	2 (0.8%)	2 (0.8%)	73 (29.2%)	18 (7.2%)	102 (40.8%)	53 (21.2%)
14	14 (5.6%)	9 (3.6%)	20 (8.0%)	44 (17.6%)	71 (28.4%)	57 (22.8%)	35 (14.0%)
15	187 (74.8%)	29 (11.6%)	16 (6.4%)	15 (6.0%)	‐	‐	3 (1.2%)
16	79 (31.6%)	43 (17.2%)	21 (8.4%)	24 (9.6%)	23 (9.2%)	22 (8.8%)	38 (15.2%)
17	6 (2.4%)	16 (6.4%)	32 (12.8%)	52 (20.8%)	26 (10.4%)	52 (20.8%)	66 (26.4%)
18	16 (6.4%)	28 (11.2%)	20 (8.0%)	53 (21.2%)	57 (22.8%)	46 (18.4%)	30 (12.0%)
19	131 (52.4%)	56 (22.4%)	24 (9.6%)	18 (7.2%)	12 (4.8%)	7 (2.8%)	2 (0.8%)
20	114 (45.6%)	41 (16.4%)	23 (9.2%)	40 (16.0%)	20 (8.0%)	8 (3.2%)	4 (1.6%)
21	‐	3 (1.2%)	2 (0.8%)	68 (27.2%)	6 (2.4%)	116 (46.4%)	55 (22.0%)
22	11 (4.4%)	30 (12.0%)	22 (8.8%)	34 (13.6%)	92 (36.8%)	56 (22.4%)	5 (2.0%)
23	207 (82.8%)	28 (11.2%)	7 (2.8%)	7 (2.8%)	‐	1 (0.4%)	‐
24	136 (54.4%)	44 (17.6%)	24 (8.4%)	21 (8.4%)	14 (5.6%)	8 (3.2%)	3 (1.2%)
25	4 (1.6%)	16 (6.4%)	21 (8.4%)	19 (7.6%)	18 (7.2%)	41 (16.4%)	131 (52.4%)
26	4 (1.6%)	9 (3.6%)	10 (4.0%)	47 (18.8%)	43 (17.2%)	73 (29.2%)	64 (25.6%)
27	222 (88.8%)	19 (7.6%)	3 (1.2%)	2 (0.8%)	‐	3 (1.2%)	1 (0.4%)
28	27 (10.8%)	25 (10.0%)	20 (8.0%)	58 (23.2%)	46 (18.4%)	44 (17.6%)	30 (12.0%)
29	5 (2.0%)	12 (4.8%)	15 (6.0%)	42 (16.8%)	30 (12.0%)	70 (28.0%)	76 (30.4%)
30	16 (6.4%)	24 (9.6%)	18 (7.2%)	48 (19.2%)	49 (19.6%)	55 (22.0%)	40 (16.0%)
31	133 (53.2%)	42 (16.8%)	21 (8.4%)	9 (3.6%)	10 (4.0%)	12 (4.8%)	23 (9.2%)
32	124 (49.6%)	39 (15.6%)	23 (9.2%)	34 (13.6%)	10 (4.0%)	12 (4.8%)	8 (3.2%)

### 4.3 Validity analyses

In the first analyse, the EFA suggested a factorial analysis of 1 to 8 elements. The KMO value for the EFA was 0.783 (*p* = 0.000) and the Bartlett's spherical test was 0.000 (*χ* ^2^ = 3,192.282; *p* = 0.000). In the 8‐factor solution, the explanatory power of the scale was 60.76%. However, according to the principle of parsimony, a second factorial analysis was performed for simulating the structure of the original scale in four factors. In this second analyse with 4‐factor solution, the explanatory power of the scale was 44.46%.

Table 3 shows items' extraction values distribution using the principal component analysis in four factors. The four factors extracted were affiliative, self‐enhancing, aggressive and self‐defeating humour. All them presented values higher than 0.30, except item 31 that showed extraction value of 0.299.

**TABLE 3:** Item loadings from rotated component matrix

Items	Factors			
	1	2	3	4
10	0.737			
6	0.693			
28	0.691			
14	0.667			
30	0.635			
26	0.608			
18	0.591			
2	0.500		0.347	
20		0.763		
24		0.762		
12		0.759		
32		0.706		
8		0.663		
4		0.520		
16		0.446		
21			0.759	
13			0.730	
17			0.654	
9			0.637	
5			0.572	
1			0.551	
22		0.307	0.540	
25			0.532	
29			0.452	
23				0,693
11				0,664
7				0,615
27				0,545
19		0.322		0,544
15				0,538
3				0,475
31				0,299
Eigen value	3.87	3.77	3.70	2.89
Variance, %	12.09	11.77	11.55	9.04
Cumulative variance, %	12.09	23.86	35.41	44.46

*Note*: Extraction method was principal axis analysis with varimax rotation.

### 4.4 Reliability analyses

Cronbach's coefficient of the developed tool was 0.82. Cronbach's values for each factor are as follows: affiliative humour 0.79, self‐enhancing humour 0.79, aggressive humour 0.64 and self‐defeating humour 0.79.

All styles of humour correlated positively and statistically significantly with each other and with the total scale score, except for aggressive humour, as shown in Table 4. Aggressive humour correlated negatively and statistically significantly with affiliative humour (*r* = −0.15; *p* = 0.02) aggressive humour did not correlate statistically significantly with self‐enhancing humour (*r* = 0.03; *p* = 0.680).

**TABLE 4:** HSQ's factor correlation

		Affiliative	Self‐enhancing	Aggressive	Self‐defeating
Self‐enhancing	*r* CI 95%	0.41** [0.26‐0.53]			
	*p*	**0.000**			
Aggressive	*r* CI 95%	‐0.15* [−0.27.‐0.03]	0.03 [−0.10‐0.15]		
	*p*	**0.020**	0.680		
Self‐defeating	*r* CI 95%	0.15* [0.03‐0.27]	0.33** [0.18‐0.47]	0.28** [0.12‐0.42]	
	*p*	**0.016**	**0.000**	**0.000**	
HSQ global	*r* CI 95%	0.60** [0.49‐0.70]	0.75** [0.67‐0.81]	0.35** [0.19‐0.48]	0.74** [0.66‐0.81]
	*p*	**0.000**	**0.000**	**0.000**	**0.000**

Abbreviations: CI, confidence interval; *p*, *p*‐value; *r*, correlation coefficient value. The Bold values indicate that p‐value was statistically significant.

* *p* < 0.05

** *p* < 0.00.

## 5 DISCUSSION

The HSQ factor structure, forced to four factors, was similar to that described in the original version and in the rest of the validation studies of the adapted questionnaire in different populations. These factors, in addition to being the most parsimonious factorial solution, explained a total variance of 44.46%, exceeding the values of the studies conducted worldwide, whose variance ranged from 34% to 42.94%, even surpassing those reported in the previous studies (Brizzio et al., 2006; Cassaretto & Martínez, 2009; Cayssials & Pérez, 2005; Chen, & Martin, 2007; Heintz & Ruch, 2015; Kazarian & Martin, 2006; Lillo, 2006; Rodríguez & Feldman, 2009; Saraglou & Scariot, 2002; Taher et al., 2008; Villareal et al., 2012). This is even higher than those reported in the original validation, with a variance of 41.60% (Martin et al., 2003). The factor structure found allows us to affirm the validity of the theoretical hypothesis of the four styles of the Sense of Humour.

In the Spanish version adapted by Cayssials & Pérez (2005), the version used in this study, confirmed the validity and reliability of the questionnaire in the Argentine population with 800 participants from the University of Buenos Aires. This structure explained 37.00% of the variance, affiliative humour explained 15.47% of the variance, self‐enhancing humour 9.20%, aggressive humour 6.30% and self‐defeating humour 6.20%. As in the present study, affiliative humour was the factor that explained the greatest percentage of variance and self‐defeating humour the least.

Cassaretto & Martínez (2009) reported a total variance of the 32 items of 39.62%, improving their explained variance to 42.94% by neutralizing 4 items for presenting problems in their factorial location, two of the self‐affirmative humour factor, item 22 *“If I feel sad or upset, I often lose my sense of humor”* and 30 “I *don't need to be with other people to have fun. I can often find things to laugh about even if I am alone”* and two others from self‐defeating humour, 16 *“I don't usually say funny things to ridicule myself”* and 28 *“If I am having problems or not feeling happy, I often cover it up by joking, so that my closest friends don't know how I really feel”*.

As in our study, item 22 presented problems in other validation studies such as Cassaretto & Martínez (2009), Villareal et al. (2012) or Brizzio et al. (2006) by placing it in the affiliative humour factor, instead of in the self‐enhancing humour factor, as indicated in the original model. Also, item 28, as in the studies by Brizzio et al. (2006), Saraglou & Scariot (2002), Chen & Martin (2007), Taher et al. (2008) and Villareal et al. (2012) presented problems, as it acquired great discriminatory power in the self‐enhancing humour factor, rather than in the self‐defeating humour factor.

Other studies have also shown problems with item 30 (Brizzio et al., 2006; Lillo, 2006; Saraglou & Scariot, 2002; Villareal et al., 2012), for not being able to discriminate between sense of humour, with item 16, which had a higher loading on the aggressive humour factor (Brizzio et al., 2006) or was neutral (Villareal et al., 2012) and with item 11 *“When I say funny things, I generally don't care how people take it”*, which had a low factor loading of 0.22 on aggressive humour (Chen & Martin, 2007). While item 31 *“Even if something is very funny to me, I will try not to laugh or joke about it, if someone might get offended*” in our study had a borderline factor loading of 0.30. In this case, it was decided to give in to this limitation in order to keep the item in the factor. The remaining items contributed at least 0.40 to the factor, as was the case in Martin's original scale (Martin et al., 2003). Lillo (2006) eliminated three items from the self‐enhancing humour factor that did not fit the saturation criteria of 0.40, resulting in a 29‐item scale.

In relation to the overall reliability coefficients, a reliability of 0.82 was obtained, similar to the levels of the original scale (Martin et al., 2003). These levels surpassed the indices of the Spanish version of Cayssials & Pérez (2005), (with a reliability of 0.75) and the rest of the studies carried out worldwide (with a reliability of around 0.63–0.80) (Cassaretto & Martínez, 2009; Chen & Martin, 2007; Heintz & Ruch, 2015; Kazarian & Martin, 2006; Lillo, 2006; Saraglou & Scariot, 2002; Taher et al., 2008). Villareal et al. (2012) exceeded the reliability level of 0.85 but eliminated items 16, 22, 28 and 30.

In terms of reliability by factors, the results of our study are in line with the original validation data (Martin et al., 2003), in the original validation study, a lower reliability was obtained for the aggressive humour factor of 0.77 with respect to the other factors, which had a Cronbach's *α* of 0.81 for the self‐enhancing humour and 0.80 for the affiliative and self‐defeating factors. In the validation study of Saraglou and Scariot (2002) and Chen and Martin (2007), the aggressive humour factor also showed lower reliability than the others. Therefore, a similar trend is reproduced in most of the current validation studies, with higher internal consistency indices for the positive humour dimensions compared to the negative ones (Cassaretto & Martínez, 2009; Chen & Martin, 2007; Heintz & Ruch, 2015; Kazarian & Martin, 2006; Rodríguez & Feldman, 2009; Saraglou & Scariot, 2002; Taher et al., 2008; Villareal et al., 2012).

As for the inter‐relationships between the humour types, they were generally low, indicating that they measured relatively different dimensions from each other, as highlighted in the original scale (Martin et al., 2003). The present study confirmed the positive correlation between the two positive types of humour, affiliative and self‐enhancing humour, which was evident in the original scale, indicating that people who used humour to improve social relationships also used self‐enhancing humour. The positive correlation between negative humours, aggressive humour and self‐defeating humour was also confirmed, as in the original scale, indicating that those who used humour in a hostile or aggressive manner also tended to use an excessively self‐defeating style.

There is controversy in the literature on the correlation between affiliative humour and aggressive humour, as in our study it was negative, indicating that people who used humour to improve social relationships did not use aggressive humour, a result that was to be expected, especially in health professionals. However, Martin et al. (2003) stated that these two humours were positively correlated, suggesting that people who frequently joke and laugh with others in order to relate to others also showed a tendency to use sarcastic or self‐enhancing humour.

Self‐defeating humour correlated positively with affiliative humour and self‐enhancing humour, indicating that people who show a tendency to use themselves as an object of humour to attract the attention of others, ridiculing or belittling themselves, do so in order to get along better socially, or as a defence coping mechanism by maintaining a humorous attitude towards the world. Martin finds no difference between these humours (Martin et al., 2003). These differences between the use of different styles of humour in different study populations may be influenced by the socio‐cultural context (Jiang et al., 2019; Kazarian & Martin, 2006; Kuiper et al., 2010; Mendiburo & Páez, 2011).

This study had certain limitations. First, the principal limitation was the low response rate of health professionals. However, based on estimations of sample size, the quality of the data remains is acceptable for the exploratory factor analysis (Carvajal et al., 2011; Kline, 1994; Lloret‐Segura et al., 2014; Nunnally & Bernstein, 1994). Nevertheless, being careful when interpreting this data as representative remains important. In fact, the sample size is insufficient for a confirmatory factor analysis. Thus, future studies should make this analysis with an adequate sample size. Second, the current study sample may not be entirely representative of all health professionals. Future studies should analyse the distinction between the styles humour and socio‐demographic variables with a sample well‐balanced.

The main strength of this study is its originality, given the scarcity of research that has aimed to identify psychometric properties of questionnaires on humour in health professionals, most of them being validated in students (Cassaretto & Martínez, 2009; Chen & Martin, 2007; Heintz & Ruch, 2015; Kazarian & Martin, 2006; Lillo, 2006; Saraglou & Scariot, 2002; Taher et al., 2008).

In conclusion, the psychometric properties of the Humour Styles Questionnaire in health professionals are similar to those reported in general population, even improved. The instrument obtained reproduces the four‐factor factor structure of the original test: affiliative humour, aggressive humour, self‐enhancing humour and self‐defeating humour. According to these results, the HSQ is a valid and reliable instrument for assessing humour in healthcare professionals.

## CONFLICT OF INTEREST

The authors declare that they have no competing interests.

## FUNDING INFORMATION

This research received no specific grant from any funding agency in the public, commercial or not‐for‐profit sectors.

## ETHICAL APPROVAL

This study was approved by the ethics committee of the Clinical investigation of Hospital Clínico San Carlos with registration number C.I.16/474‐E.

## Data Availability

The data that support the findings of this study are available on request from the corresponding author. The data are not publicly available due to privacy or ethical restrictions.
